# TCP Transcription Factors Associate with PHYTOCHROME INTERACTING FACTOR 4 and CRYPTOCHROME 1 to Regulate Thermomorphogenesis in *Arabidopsis thaliana*

**DOI:** 10.1016/j.isci.2019.04.002

**Published:** 2019-05-08

**Authors:** Yu Zhou, Qingqing Xun, Dongzhi Zhang, Minghui Lv, Yang Ou, Jia Li

**Affiliations:** 1 Ministry of Education Key Laboratory of Cell Activities and Stress Adaptations, School of Life Sciences, Lanzhou University, Lanzhou 730000, P.R. China

**Keywords:** Biological Sciences, Molecular Biology, Plant Biology

## Abstract

Temperature, one of the most critical environmental cues, greatly affects plant growth, development, and reproduction. PHYTOCHROME-INTERACTING FACTOR 4 (PIF4), a key transcription factor in light signaling pathway, plays a central role in temperature-mediated growth responses. How higher temperature regulates the function of PIF4, however, is not well understood. Here we demonstrate that three phylogenetically related TEOSINTE BRANCHED 1/CYCLOIDEA/PCF (TCP) transcription factors, TCP5, TCP13, and TCP17, play fundamental roles in promoting thermoresponsive hypocotyl growth by positively regulating the activity of PIF4. TCP17 was found to interact with a blue light receptor, CRYPTOCHROME 1 (CRY1), at lower temperature, leading to reduced activity of TCP17. Higher temperature can increase the stability of TCP17, and release TCP17 from the CRY1-TCP17 complex, allowing it to upregulate the expression of *PIF4* and enhance the transcriptional activity of PIF4. This study revealed the important roles of TCPs in regulating the activity of PIF4 in thermomorphogenesis.

## Highlights

• TCP transcription factors promote PIF4-mediated thermoresponsive hypocotyl growth
• TCP17 interacts with PIF4 to promote the transcriptional activity of PIF4
• CRY1 interacts with TCP17 and represses the binding affinity of TCP17 with PIF4
• Higher temperature releases TCP17 from the repression of CRY1

## Graphical Abstract

**Figure:**
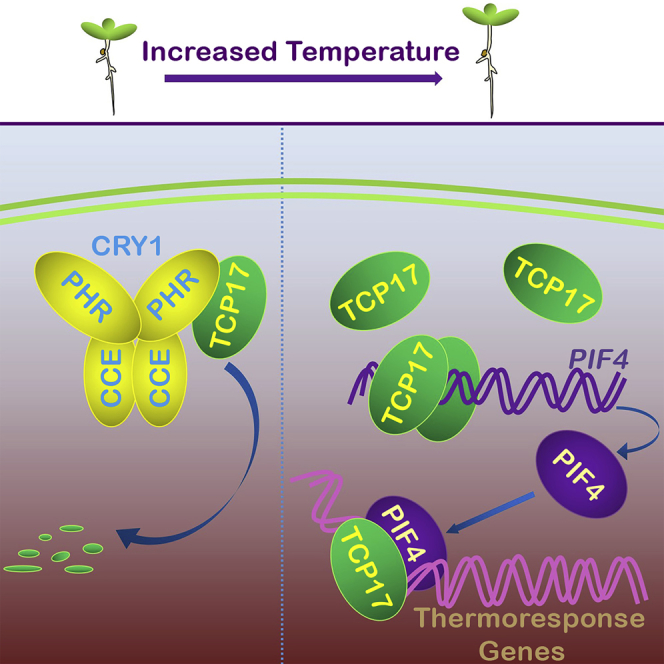

## Introduction

Plants are sessile in nature, growth and development of which have to coordinate with their ever-changing living environments for better survival and reproduction (Lau and Deng, 2010, McClung et al., 2016). In addition to light, water, and nutrients, ambient temperature is another key environmental factor regulating multiple physiological processes in the life cycle of a plant (McClung et al., 2016, Quint et al., 2016, Vert and Chory, 2011, Wigge, 2013). Elevated temperature can cause a series of morphological changes of a plant, including elongated hypocotyls, early flowering, and reduced reproduction (McClung et al., 2016, Quint et al., 2016, Wigge, 2013).

PHYTOCHROME-INTERACTING FACTOR 4 (PIF4), a key regulator in light signal transduction (Castillon et al., 2007, Huq and Quail, 2002, Leivar and Monte, 2014, Leivar and Quail, 2011), also acts as a central hub in a thermoresponsive pathway (Koini et al., 2009, Wigge, 2013). PIF4 integrates with several endogenous growth-regulating phytohormones, including auxin, gibberellins, and brassinosteroids, to mediate the expression of a series of high-temperature responsive genes (Franklin et al., 2011, Koini et al., 2009, Oh et al., 2012, Stavang et al., 2009, Sun et al., 2012). Owing to the critical roles of PIF4 in connecting environmental signals to endogenous responses, its function is tightly regulated (Leivar and Monte, 2014, Leivar and Quail, 2011).

Recently, a red/far-red light photoreceptor PHYTOCHROME B (PHYB) was proposed as a temperature sensor in *Arabidopsis* (Jung et al., 2016, Legris et al., 2016). Elevated ambient temperature signal can be perceived by PHYB, turning PHYB from its bioactive form (Pfr) to an inactive form (Pr) (Jung et al., 2016, Legris et al., 2016). Pfr physically associates with PIF4 and blocks its transcription activity. However, Pr cannot interact with PIF4, allowing PIF4 to upregulate the expression of thermoresponsive genes, promoting hypocotyl growth at high ambient temperatures (Jung et al., 2016, Legris et al., 2016). In addition to PHYB, CRYPTOCHROME 1 (CRY1), a photolyase-like blue light receptor originally isolated from *Arabidopsis* (Briggs and Huala, 1999, Lin, 2002, Sancar, 2003, Sancar et al., 2000), which inhibits hypocotyl elongation in blue light by forming a complex with SUPPRESSOR OF PHYA-105 (SPA1) and CONSTITUTIVE PHOTOMORPHOGENIC 1 (COP1) (Deng et al., 1991, Lian et al., 2011, Liu et al., 2011), was reported to regulate thermoresponsive hypocotyl growth by inhibiting the transcriptional level of *PIF4* and interacting with PIF4 in blue light to suppress the activity of PIF4, especially at an elevated temperature (Ma et al., 2016).

Despite the critical role of photoreceptors in temperature sensing, multiple components in photomorphogenesis and circadian rhythm were also found to regulate the activity of PIF4 in thermomorphogenesis. DE-ETIOLATED 1 (DET1) and COP1 were demonstrated to regulate high-temperature-induced growth by promoting *PIF4* transcript abundance through ELONGATED HYPOCOTYL 5 (HY5) (Delker et al., 2014). Besides the DET1/COP1-HY5 cascade in regulating the expression of *PIF4*, there are also distinct mechanisms between DET1/COP1 and HY5 in regulating hypocotyl growth at high temperatures. DET1/COP1 complex is necessary for upregulating *PIF4* expression and stability of PIF4 (Gangappa and Kumar, 2017), whereas HY5 competes with PIF4 for G-box motifs in the promoters of its target genes (Gangappa and Kumar, 2017, Toledo-Ortiz et al., 2014). EARLY FLOWERING 3 (ELF3), an important component of evening complex of circadian clock, was found to suppress the transcription levels of *PIF4* and *PIF5* (Nusinow et al., 2011). ELF3 interacts with PIF4 and blocks the role of PIF4 in activating the expression of thermoresponsive genes (Box et al., 2015, Nieto et al., 2015). In a recent study, TOC1/PRR5, another key component in circadian clock, was revealed to interact with PIF4, inhibiting circadian gating of PIF4 in thermomorphogenesis (Zhu et al., 2016). FLOWERING TIME CONTROL PROTEIN, an RNA-binding protein, acts as another important factor in regulating temperature-mediated flowering and hypocotyl growth by suppressing the activity of PIF4 (Blazquez et al., 2003, Lee et al., 2014, Macknight et al., 1997).

Given the fact that many factors have been proposed to interact with PIF4 and inhibit its activity, regulatory components positively regulating the activity of PIF4 in thermoresponses, however, are poorly understood. In this study, we demonstrated that TCP transcription factors positively regulate thermoresponsive hypocotyl elongation by increasing *PIF4* expression and the transcriptional activity of PIF4. High temperature increases the transcriptional activity of TCP17 toward *PIF4* and the interaction between TCP17 and PIF4 by relieving the repression of TCP17 from CRY1. Our studies reveal a novel molecular mechanism of TCPs in integrating the functions of CRY1 and PIF4 to regulate hypocotyl growth at high ambient temperatures.

## Results

### TCPs Positively Regulate Thermomorphogenesis

**Figure 1:**
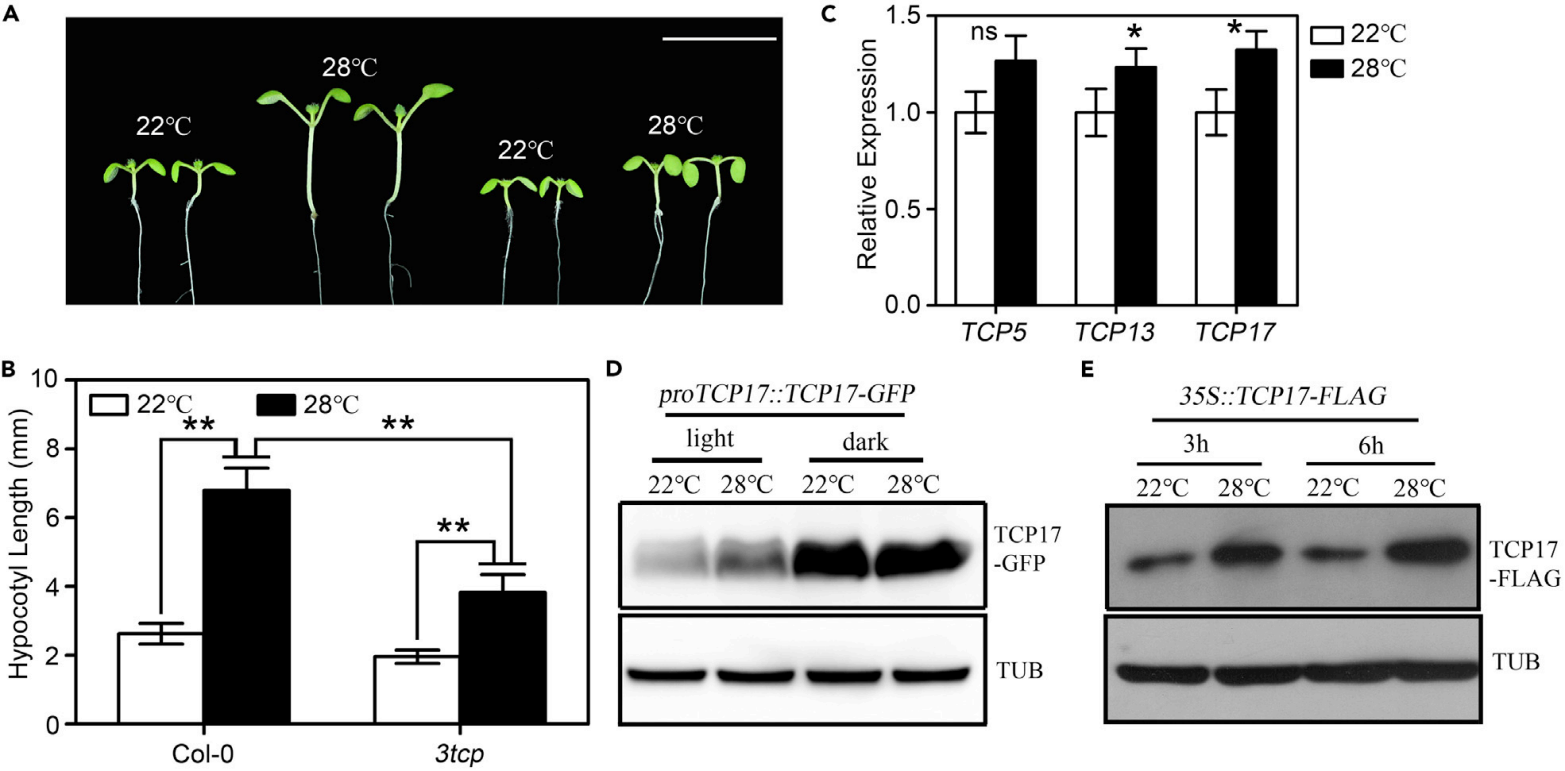
TCPs Act as Positive Regulators in Promoting Thermomorphogenesis (A and B) *tcp5 tcp13 tcp17* (*3tcp*) triple mutant shows a reduced hypocotyl elongation phenotype under higher temperature. Phenotypes (A) and hypocotyl measurements (B) of wild-type and *3tcp* seedlings grown at 22°C or 28°C. Scale bars, 1 cm. Data shown are the average and SEM of three independent biological replicates (n ≥ 20 for each replicate). **p < 0.01; based on Student's t test. (C) The transcriptional levels of *TCP5*, *TCP13*, and *TCP17* are slightly increased after high-temperature treatment. Seven-day-old Col-0 seedlings grown under LD 22°C were transferred to 28°C or remained at 22°C for 4 h before being collected for real time RT-PCR analysis. Data are represented as mean ± SEM. ns p ≥ 0.05, and *p < 0.05; based on Student's t test. (D) The responses of TCP17 protein from *proTCP17::TCP17-GFP* plants to elevated temperature. Seedlings were grown under LD at 22°C for 7 days, and then half of them were transferred to 28°C at ZT-12 or ZT-20 for 4 h. The levels of TCP17-GFP and tubulin were detected by an anti-GFP or an anti-tubulin (TUB) antibody, respectively. (E) The responses of TCP17 protein from *35S::TCP17-FLAG* plants to elevated temperature. Seedlings were grown at LD at 22°C for 7 days, and then were treated at 22°C or 28°C for 3 or 6 h. An anti-FLAG or anti-tubulin antibody was used for detecting the accumulation of TCP17-FLAG and tubulin.

High-temperature-induced morphological changes are reminiscent of what is seen in a shade condition, suggesting a possibly common molecular mechanism between these two signaling pathways (Legris et al., 2017, Quint et al., 2016). Our previous studies revealed that three phylogenetically related TCP transcription factors, TCP5, TCP13, and TCP17, play a crucial role in promoting hypocotyl elongation in shade (Zhou et al., 2018). To investigate whether these TCPs are also required for thermoresponsive hypocotyl growth, we analyzed the hypocotyl responses of loss- or gain-of-function mutants of these three *TCPs* to elevated temperature. We found that the hypocotyl growth responses of *tcp5*, *tcp13*, or *tcp17* single mutant to higher temperature are similar to those of Col-0 (Figures S1A and S1B). However, a *tcp5 tcp17* double mutant showed a significantly impaired hypocotyl response to elevated temperature (Figures S1A and S1B) and the thermoresponsive defect of the *tcp5 tcp13 tcp17* triple mutant, *3tcp*, is more significant than that of the *tcp5 tcp17* double mutant (Figures 1A, 1B, S1A, and S1B). In contrast, transgenic seedlings overexpressing *TCP5*, *TCP13*, or *TCP17* showed greatly elongated hypocotyls even at 22°C, the optimal *Arabidopsis* growth temperature in a laboratory condition, indicating constitutive thermomorphogenesis (Figures S1A and S1B). These results suggested a redundant role of TCP5, TCP13, and TCP17 in promoting thermomorphogenesis.

To understand whether the expressions of *TCP*s are regulated by temperature, we investigated the transcriptional responses of *TCP5*, *TCP13*, and *TCP17* to a higher-temperature treatment in wild-type seedlings. Seven-day-old Col-0 seedlings grown at 22°C and a long-day (LD, 16-h light/8-h dark) condition were transferred to 28°C or kept at 22°C for 4 h before collected for RNA extraction. We examined the mRNA levels of *TCP5*, *TCP13*, and *TCP17* by a real-time PCR assay and observed that the transcriptional levels of these *TCP*s were slightly increased after a higher temperature treatment compared with those under 22°C (Figure 1C). Our previous studies demonstrated that TCP17 is an unstable protein in light, and its stability can be dramatically increased by shade treatment (Zhou et al., 2018). We therefore tested whether higher temperature can also affect the protein stability of TCP17. Seedlings of a representative homozygous *proTCP17::TCP17-GFP* transgenic line were grown under LD at 22°C condition for 7 days, and half of them were transferred to and kept at 28°C from ZT-12 (zeitgeber time 12) to ZT-16 (light) or ZT-20 to ZT-24 (dark) before being collected for protein extraction. Our immunoblotting results showed that TCP17-GFP was greatly accumulated in the dark, and that higher temperature has no obvious effect on the additional accumulation of TCP17 (Figure 1D). However, higher temperature can significantly elevate the protein level of TCP17 in the light (Figure 1C). To exclude the impact of the transcription of *TCP17*, protein analyses were conducted by using transgenic plants from a representative transgenic line constitutively expressing TCP17-FLAG (*35S::TCP17-FLAG*). Our immunoblotting results showed that the TCP17-FLAG level was greatly increased after transferring 22°C-grown *35S::TCP17-FLAG* seedlings to 28°C for additional 3 or 6 h (Figure 1E). In contrast, the accumulation of TCP17-FLAG was significantly decreased after transferring 28°C-pretreated seedlings to 22°C for indicated time periods (Figure S1C). We also found that the degradation of TCP17 at 22°C was significantly suppressed by the treatment of MG132 (Figure S1D), suggesting the contribution of a 26S proteasome pathway to the instability of TCP17 at 22°C. Our results indicated that higher temperature increases the protein stability of TCP17, allowing it to be accumulated in the nucleus and promote thermomorphogenesis.

### PIF4 Is Essential for TCP17 to Promote Hypocotyl Growth at Higher Temperature

**Figure 2:**
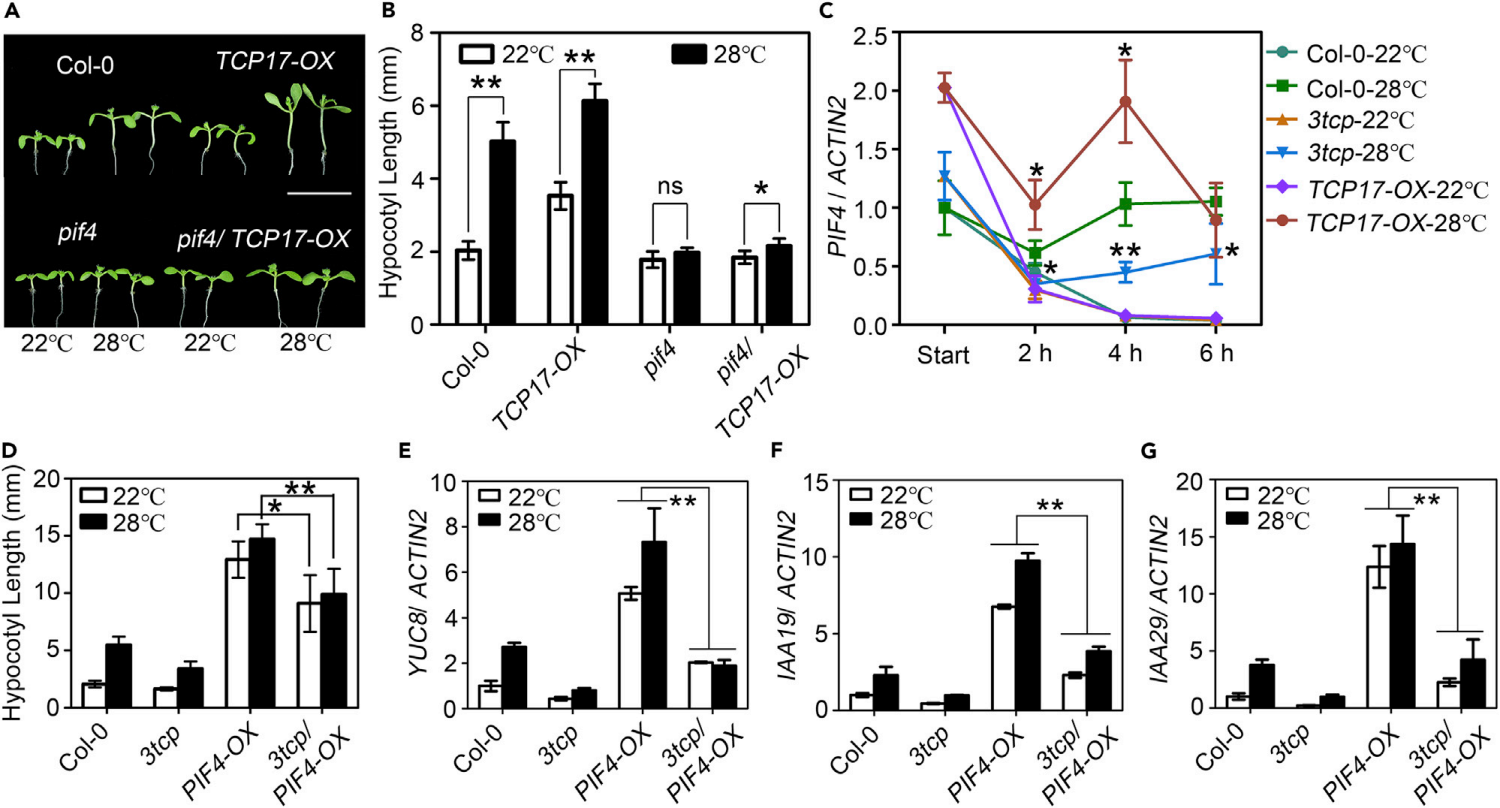
TCP17 Promotes Thermoresponsive Hypocotyl Growth via PIF4 (A and B) Phenotypes (A) and hypocotyl measurements (B) of Col-0, *TCP17-OX* (*35S::TCP17-FLAG*), *pif4*, and *pif4/TCP17-OX* grown at 22°C or 28°C. Scale bars, 1 cm. Data shown are the average and SEM of three independent biological replicates. ns p ≥ 0.05, *p < 0.05, and **p < 0.01; based on Student's t test. (C) The expression level of *PIF4* from Col-0, *TCP17-OX*, and *3tcp* in response to elevated temperature. Seedlings were grown under LD at 22°C condition for 7 days, and half of them were transferred to 28°C at ZT-18 for 2, 4, or 6 h. Whole seedlings were collected for RNA extraction at ZT-20, ZT-22, or ZT-24. Data are represented as mean ± SEM. *p < 0.05 and **p < 0.01. Student's t tests were used for the statistical analyses, showing the comparison of *TCP17-OX*-28°C or *3tcp*-28°C with Col-0-28°C. (D) The hypocotyl responses of Col-0, *3tcp*, *PIF4-OX* (*35S::PIF4-FLAG*), and *3tcp*/*PIF4-OX* to high temperature. Data are represented as mean ± SEM. *p < 0.05 and **p < 0.01; based on Student's t test. (E–G) The expression of *YUC8* (E), *IAA19* (F), and *IAA29* (G) in Col-0, *3tcp*, *PIF4-OX*, and *3tcp/PIF4-OX* plants under 22°C and 28°C. Seven-day-old seedlings grown at 22°C were transferred to 28°C or kept at 22°C for 4 h before being collected for real-time PCR analyses. Data shown are the average and SEM of three independent biological replicates. *p < 0.05 and **p < 0.01; based on the Student's t test. In (A), (B), and (D) seedlings were grown under LD at 22°C condition for 5 days and then were transferred to 28°C or kept at 22°C for additional 3 days before the picture and measurements were taken. n ≥ 20 for each replicate.

Previous studies demonstrated that PIF4 acts as a key factor in regulating thermoresponsive hypocotyl growth (Wigge, 2013). To reveal whether TCP17 promotes thermoresponsive hypocotyl growth by regulating the function of PIF4, genetic and biochemical analyses were carried out to investigate the interaction between PIF4 and TCPs. We generated *pif4/35S::TCP17-FLAG* (*pif4/TCP17-OX*) plants by crossing *pif4* with the representative transgenic line of *35S::TCP17-FLAG* (*TCP17-OX*). The obtained *pif4/TCP17-OX* seedlings displayed significantly reduced hypocotyl elongation at 22°C compared with the *TCP17-OX* transgenic seedlings (Figures 2A and 2B). In addition, the thermoresponse of *pif4/TCP17-OX* seedlings was greatly impaired, showing a response similar to that of the *pif4* mutant (Figures 2A and 2B). Consistently, the results of real-time RT-PCR analyses showed that the expression levels of several known PIF4 target genes, *YUC8*, *IAA19*, and *IAA29* (Ma et al., 2016), were significantly increased in *TCP17-OX* transgenic seedlings, whereas they were decreased in *3tcp* (Figures S2A–S2C). Furthermore, the responses of *YUC8*, *IAA19*, and *IAA29* to elevated temperature were greatly impaired in *3tcp* (Figures S2A–S2C). As a central regulator of thermomorphogenesis, PIF4 promotes hypocotyl elongation at higher temperature by increasing the expression of *YUC8* and *TAA1* (Franklin et al., 2011, Sun et al., 2012), whose encoded proteins are key enzymes catalyzing free indole-3-acetic acid biosynthesis (Tao et al., 2008, Zhao, 2010, Zhao et al., 2001). Consistently, *pif4* showed diminished higher-temperature-induced auxin accumulation and hypocotyl growth (Franklin et al., 2011, Koini et al., 2009, Sun et al., 2012). Our hypocotyl analyses showed that exogenous treatment of picloram, an analog of auxin, can significantly rescue hypocotyl growth of *3tcp* (Figures S2D and S2E), indicating that the PIF4-auxin cascade is required for TCP17-promoted thermoresponsive hypocotyl elongation. These results demonstrated that TCP17-induced thermoresponsive hypocotyl growth is largely dependent on the function of PIF4.

### TCPs Regulate Thermomorphogenesis by Promoting *PIF4* Expression and the Transcriptional Activity of PIF4

Our previous studies demonstrated that TCP17 can directly bind to the promoter of *PIF4* to elevate its expression during shade avoidance (Zhou et al., 2018). To determine whether TCPs can promote the expression of *PIF4* in response to high temperature, we analyzed the mRNA levels of *PIF4* in Col-0, *3tcp*, and *TCP17-OX* seedlings after treatment with higher temperature for different time periods. Our real-time RT-PCR analyses showed that at 28°C, the expression of *PIF4* was significantly increased in *TCP17-OX* transgenic plants and decreased in *3tcp*, and the response of *PIF4* expression to high temperature was greatly impaired in *3tcp* (Figure 2C). These results indicated that high-temperature-mediated upregulation of *PIF4* is partially via TCP transcription factors. We also investigated whether PIF4 regulates the expression of *TCP*s in response to elevated temperature. The results from real-time RT-PCR showed that, compared with wild-type, the expression levels of *TCP5* and *TCP17* from *pif4* were significantly reduced at 28°C (Figure S2F). In addition, the expression of these three *TCP*s were greatly increased in *PIF4-OX* (*35S::PIF4-FLAG*) transgenic plants (Figure S2F). These results indicated that the expression of *TCP*s can also be regulated by PIF4 in thermoresponses.

As a key factor mediating ambient temperature response, PIF4 can be regulated at multiple levels (Quint et al., 2016, Wigge, 2013). In addition to increasing the expression of *PIF4*, high temperature also can impact the transcriptional activity of PIF4 (Quint et al., 2016, Wigge, 2013). To examine whether TCPs are involved in promoting the transcriptional activity of PIF4, we analyzed the hypocotyl responses of Col-0, *3tcp*, *PIF4-OX*, and *3tcp/PIF4-OX* to higher-temperature treatment. *PIF4-OX* transgenic plants showed extremely elongated hypocotyls compared with Col-0, whereas the hypocotyls of *3tcp/PIF4-OX* seedlings were much shorter than those of *PIF4-OX* seedlings (Figure 2D). Consistently, our real-time PCR analyses showed that the expression levels of PIF4 target genes, *YUC8*, *IAA19*, and *IAA29*, were dramatically elevated in *PIF4-OX* plants compared with Col-0 plants (Figures 2E–2G). The expression of these three genes in *3tcp/PIF4-OX* plants, however, was significantly impaired, especially at higher temperature, compared with that in *PIF4-OX* plants (Figures 2E–2G). Our genetic and molecular data indicated that TCPs are required for PIF4-promoted thermoresponses.

### High Temperature Enhances the Interaction between TCP17 and PIF4 to Increase the Transcriptional Activity of PIF4

**Figure 3:**
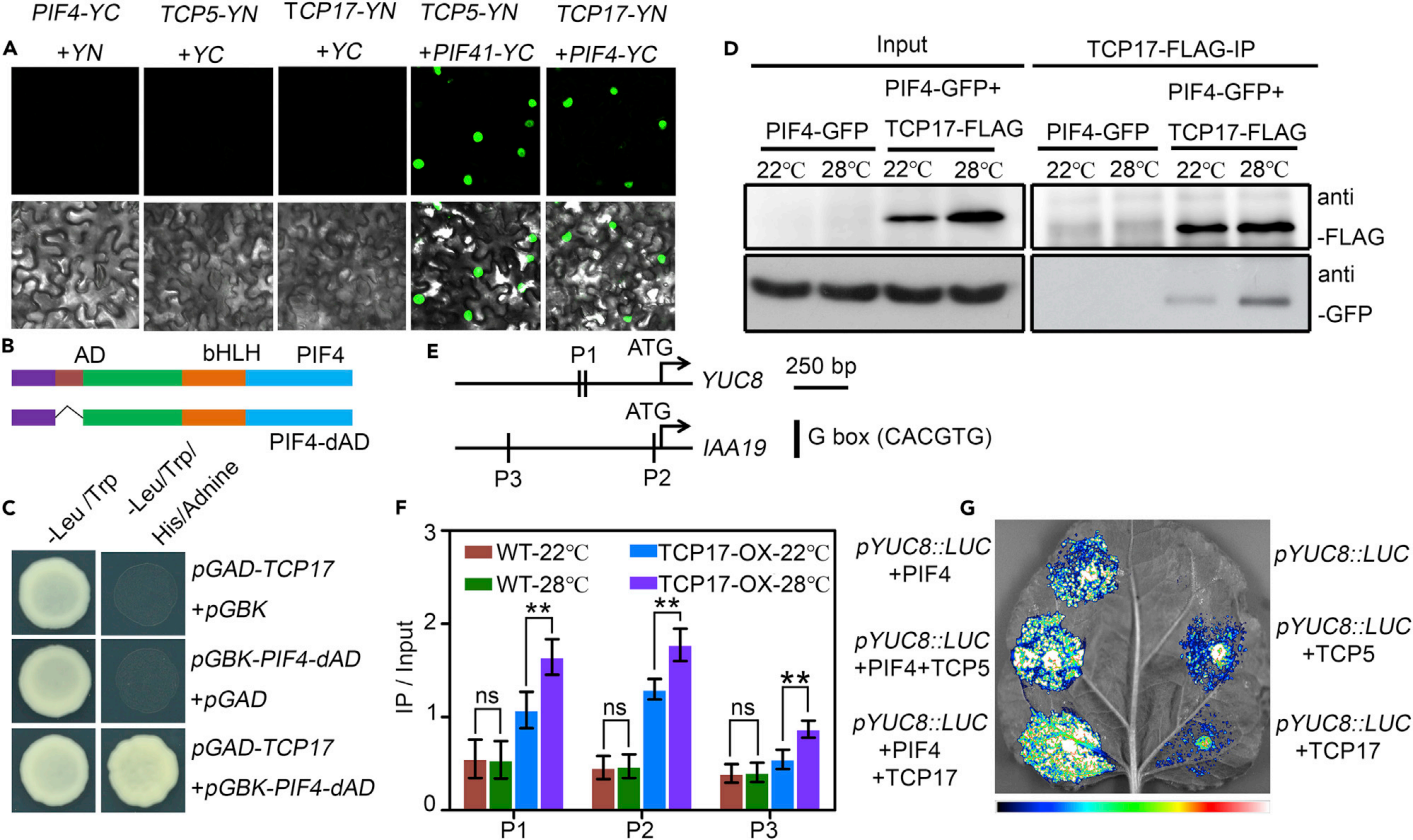
TCP17 Interacts with PIF4 in a Temperature-Dependent Manner and Promotes the Transcriptional Activity of PIF4 (A) BIFC assay shows that both TCP5 and TCP17 can interact with PIF4 in the nucleus. *Nicotiana benthamiana* leaves were co-infiltrated with *PIF4-YC* and *YN*, *TCP5-YN* and *YC*, *TCP17-YN* and *YC*, *TCP5-YN* and *PIF4-YC*, and *TCP17-YN* and *PIF4-YC*, respectively. *YN* (*nYFP*); *YC* (*cYFP*). (B) Box diagrams of full-length PIF4 and an activation domain (AD) deletion fragment of PIF4 (PIF4-dAD) used in a yeast two-hybrid analysis shown in (C). (C) Direct interaction between TCP17 and PIF4-dAD can be detected via a yeast two-hybrid assay. Yeast clones were grown on synthetic double dropout medium (-Leu/Trp) or synthetic triple dropout medium (-Leu/Trp/His) without adenine. (D) coIP assays showed that high temperature promotes the interaction between TCP17 and PIF4. (E) Schematic diagrams showing the presence of G boxes in the promoters of *YUC8* and *IAA19*. P1, P2, and P3 represent primers used in (F). (F) ChIP assays showed that high temperature promotes TCP17 to bind to the promoters of *YUC8* and *IAA19*, by using the primers flanking the G boxes as indicated in (E). Data shown are the average and SD. ns p ≥ 0.05, and **p < 0.01. Student's t tests were used for the statistical analyses. (G) TCP5 and TCP17 increased the activity of PIF4 as revealed in a transient assay. *N. benthamiana* leaves were co-infiltrated with the *pYUC8::LUC* reporter and the effectors (*35S::HA-TCP5*, *35S::TCP17-FLAG*, *35S::PIF4-GFP*, *35S::PIF4-GFP* and *35S::HA-TCP5* together, or *35S::PIF4-GFP* and *35S::TCP17-FLAG* together). Forty-eight hours after infiltration, the luciferase activities were imaged using a Lumazone CA 1300B camera.

To determine whether TCPs promote the transcriptional activity of PIF4 via direct interaction with PIF4, we investigated the physical interaction between TCPs and PIF4 *in vitro* and *in vivo*. We detected the interaction between TCP17 and PIF4 *in planta* by a bimolecular fluorescence complementation (BIFC) assay. As shown in Figure 3A, strong fluorescence was observed in the nuclei of *Nicotiana benthamiana* leaf cells co-infiltrated with *Agrobacterium* harboring *TCP17-cYFP* and *PIF4-nYFP* plasmids. Interaction between TCP5 and PIF4 was also observed (Figure 3A). We further verified the interaction between TCP17 and PIF4 using a yeast two-hybrid system. Because of high auto-activation of PIF4, the activation domain (AD) at the N terminus of PIF4 was deleted (PIF4-dAD) before being cloned into a bait vector (Figure 3B). Our results indicated that TCP17 physically interacts with PIF4 in yeast (Figure 3C). The *in vivo* interaction between PIF4 and TCP17 was confirmed by a co-immunoprecipitation (coIP) assay. We found that PIF4 was co-immunoprecipitated with TCP17 from plants at 22°C (Figure 3D). Such coIP was significantly increased upon higher-temperature treatment (Figure 3D).

Consistent with the results that higher temperature can promote the direct interaction between TCP17 and PIF4, our chromatin immunoprecipitation (ChIP) followed by real-time RT-PCR using the aforementioned *35S::TCP17-FLAG* (*TCP17-OX*) transgenic plants showed that TCP17 can associate with the G-box-motif-containing regions in the promoters of *YUC8*, and *IAA19* that PIF4 binds to (Figures 3E and 3F). The association between TCP17 and the promoter regions of *YUC8* and *IAA19* was greatly enhanced by higher temperature (Figure 3F). To further investigate whether TCP17 affects the transcription activity of PIF4, a transient transcription assay was carried out to analyze the effects of TCPs on PIF4 transcription activities, by using a firefly luciferase (*LUC*) gene driven by the promoter of *YUC8* (*pYUC8::LUC*) as a reporter system. Co-infiltration analysis in *N. benthamiana* leaves indicated that co-expression of *PIF4* and *TCP5*, or *PIF4* and *TCP17*, can drastically increase the expression of *LUC*, when compared with the one only expressing *PIF4* (Figure 3G). These results proved that TCP17 forms a complex with PIF4 *in vitro* and *in vivo*, and the interaction was significantly increased by higher temperature, leading to significantly enhanced transcription activity of PIF4.

### TCPs Are Involved in CRY1-Mediated Thermomorphogenesis

Previous studies demonstrated that a blue light receptor CRY1 can interact with PIF4 in a blue-light-dependent manner to repress the transcription activity of PIF4 and growth responses to elevated temperature (Ma et al., 2016). However, mechanisms by which CRY1 regulates the activity of PIF4 in response to temperature changes are not well understood. In addition, transcription factors interacting with CRY1 to regulate the expression of *PIF4* in thermoresponse remain elusive.

**Figure 4:**
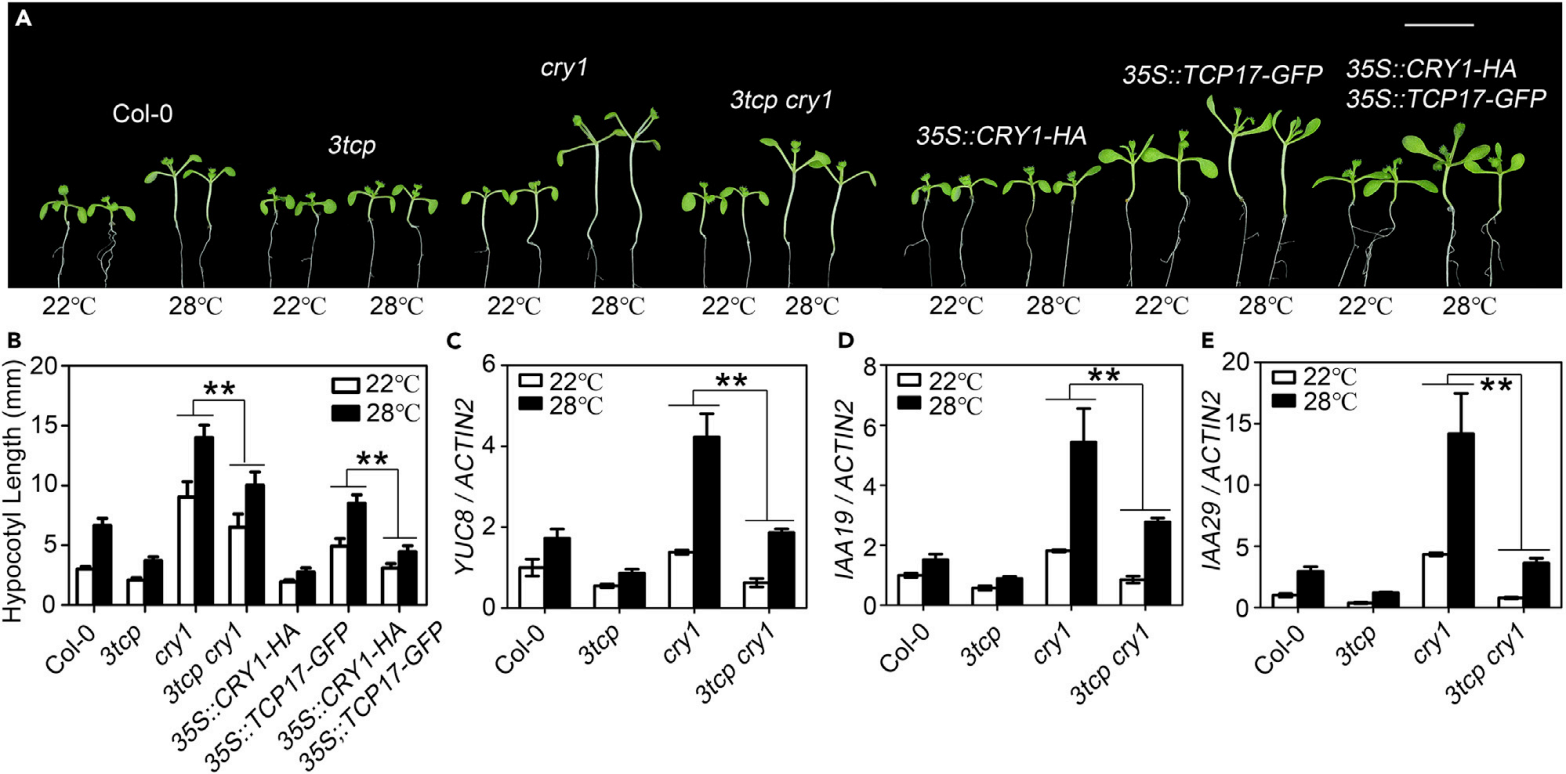
TCPs Play Important Role in CRY1-Mediated Thermoresponsive Hypocotyl Growth (A and B) Genetic interaction between CRY1 and TCPs. Hypocotyl phenotypes (A) and measurements (B) of Col-0, *3tcp*, *cry1*, *3tcp cry1*, *35S::CRY1 -HA*, *35S::TCP17-GFP*, and *35S::CRY1-HA*/*35S::TCP17-GFP* grown at 22°C or 28°C. Scale bars, 1 cm, and n ≥ 20 for each replicate. (C–E) The expression of PIF4 target genes *YUC8* (C), *IAA19* (D), and *IAA29* (E) in Col-0, *3tcp*, *cry1*, and *3tcp cry1* at 22°C and 28°C. Seedlings were grown under LD condition at 22°C for 7 days were transferred to 28°C or remained at 22°C for 4 h before being collected for real-time PCR analysis. In (B–E), data shown are the average and SEM of three independent biological replicates. **p < 0.01. Student's t tests were used for the statistical analyses.

To reveal whether TCPs are required for CRY1-mediated thermoresponsive hypocotyl growth, we examined the genetic interaction between *TCPs* and *CRY1*. The hypocotyl responses of Col-0, *3tcp*, *cry1*, *3tcp cry1, 35S::CRY1-HA, 35S::TCP17-GFP,* and *35S::CRY1-HA/35S::TCP17-GFP* to elevated temperature were analyzed. Consistent with the results from a previous study (Ma et al., 2016), *cry1* showed dramatically elongated hypocotyls at both 22°C and 28°C; the hypocotyl length of *3tcp cry1*, however, was greatly reduced compared with *cry1* (Figures 4A and 4B). In addition, the hypocotyls of *35S::CRY1-HA* transgenic seedlings showed a greatly reduced response to higher temperature, and TCP17-induced hypocotyl elongation at high temperature was significantly impaired in the *35S::CRY1-HA* background (Figures 4A and 4B). Consistently, our real-time RT-PCR analyses showed that the expressions of PIF4-targeted genes, *YUC8*, *IAA19*, and *IAA29* from *cry1* are much higher than that from Col-0, whereas the transcriptional levels of these genes are greatly impaired in the *3tcp cry1* quadruple mutant compared with *cry1* (Figures 4C–4E). Our genetic and molecular data strongly demonstrated that CRY1 inhibits thermomorphogenesis partially via repressing the functions of TCPs.

### CRY1 Physically Interacts with TCP17 in a Temperature-Dependent Manner

**Figure 5:**
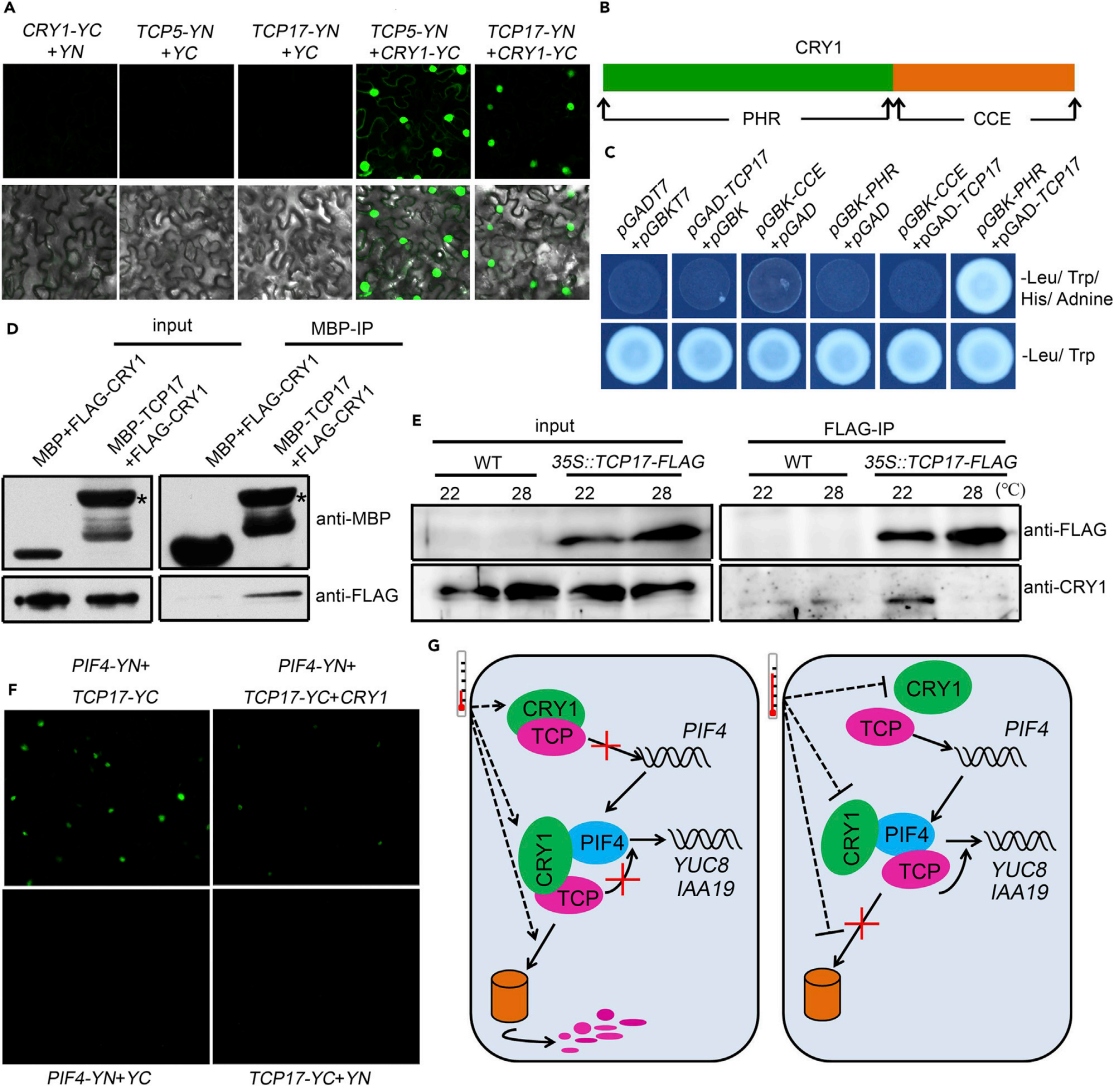
CRY1 Forms a Complex with TCP17 at Lower Temperature to Repress the Interaction between TCP17 and PIF4 (A) BIFC assays in *Nicotiana benthamiana* leaves showed strong interactions between CRY1 and TCP5 or TCP17. (B) The schematic diagram shows the PHR (N-terminal photolyase-related domain) and CCE (C-terminal extension domain) domains of CRY1 protein. (C) Yeast two-hybrid assays showed an interaction between TCP17 and the PHR domain of CRY1. (D) Interaction of TCP17 and CRY1 can be detected in an *in vitro* pull-down assay. *E. coli* expressed and purified MBP and MBP-tagged TCP17 were incubated with FLAG-CRY1 for 2 h at 4°C; the products were analyzed by immunoblotting with anti-MBP or anti-FLAG antibody. * represented MBP-TCP17. (E) TCP17 interacts with CRY1 in a temperature-dependent manner *in vivo*. An anti-FLAG affinity matrix (Sigma) was used for immunoprecipitation analyses. The input and the immunoprecipitation (IP) products were probed by an anti-FLAG or an anti-CRY1 antibody. (F) BIFC assay showed that the interaction between PIF4 and TCP17 in *N. benthamiana* leaves is inhibited by co-expression of *35S::CRY1-HA*. (G) A hypothetical mechanism by which TCP transcription factors regulates thermomorphogenesis.

To determine whether these TCPs are involved in CRY1-regulated thermomorphogenesis by directly interacting with CRY1, we investigated the physical interaction between CRY1 and TCPs *in vitro* and *in vivo*. In a BIFC assay, strong fluorescence was observed in the nucleus of *N. benthamiana* leaf cells after co-infiltration with *Agrobacterium* mixtures harboring *CRY1-cYFP* and *TCP17-nYFP* or *CRY1-cYFP* and *TCP5-nYFP* plasmids (Figure 5A). Also, we tested the interaction between TCP17 and CRY1 in a yeast two-hybrid system. *Arabidopsis* CRY1 is a photolyase-like blue light receptor (Briggs and Huala, 1999, Lin, 2002). CRY1 contains two functional domains, an N-terminal photolyase-related (PHR) domain for chromophore binding and a C-terminal extension (CCE) domain for protein-protein interactions (Figure 5B) (Yu et al., 2010). Because of strong autoactivation of full-length CRY1 protein, we tested the interaction between TCP17 and PHR or CCE domain and found that TCP17 interacts strongly with PHR, but not with CCE domain (Figure 5C). Consistently, *E. coli*-purified TCP17 and CRY1 also showed interaction in an *in vitro* pull-down assay (Figure 5D). More remarkably, the interaction between CRY1 and TCP17 in *Arabidopsis* showed temperature dependence. CRY1 was co-immunoprecipitated with TCP17 from plants grown at 22°C, but the interaction was greatly reduced at 28°C (Figure 5E).

### CRY1 Inhibits the Activity of TCP17 in Promoting *PIF4* Expression and the Interaction between TCP17 and PIF4

The temperature-dependent interaction between TCP17 and CRY1 suggests a fundamental role of CRY1 in regulating the function of TCP17 in thermomorphogenesis. Our results showed that the stability of TCP17 protein is regulated by temperature. To investigate whether the degradation of TCP17 at low temperature is mediated by CRY1, we tested the response of TCP17 protein in *cry1* or *35S::CRY1-HA* background to different temperatures. Our immunoblotting analysis showed that the level of TCP17 protein from *35S::CRY-HA* or *cry1* in response to temperature changes was not significantly altered compared with that from Col-0 (Figure S3A). This result suggested that the degradation of TCP17 at lower temperature is not caused by the interaction between CRY1 and TCP17. The detailed mechanism by which temperature regulates the stability of TCP17 needs to be clarified in the future.

Previous studies demonstrated that a CRY1 loss-of-function mutant can greatly elevate the expression of *PIF4* in response to high temperature (Ma et al., 2016). To reveal whether CRY1 is involved in TCP-mediated regulation of *PIF4* expression, we examined the responses of *PIF4* from *cry1* and *3tcp cry1* to elevated temperature. Our data showed that the expression of *PIF4* from *cry1* was much higher than that from Col-0, whereas the *PIF4* expression from *3tcp cry1* in response to high temperature was significantly reduced compared with that from *cry1* (Figure S3B). Our further transient transcription assay in *N. benthamiana* by using a *pPIF4::LUC* reporter system showed that CRY1 can significantly reduce the transcriptional activity of TCPs toward *PIF4* (Figure S3C). These data indicated that CRY1 negatively regulates *PIF4* expression partially by repressing the activity of TCPs.

Considering the results that high temperature releases TCP17 from the TCP17-CRY1 complex (Figure 5E), and increases the interaction between TCP17 and PIF4 (Figure 3D), we hypothesized that CRY1 forms a complex with TCP17 to suppress the interaction between TCP17 and PIF4. To investigate whether CRY1 affects the interaction between TCP17 and PIF4, we transiently expressed *TCP17-nYFP* and *PIF4-cYFP* with or without *CRY1* in *N. benthamiana* leaves. Consistently, strong fluorescence was observed in the cells co-expressing *TCP17-nYFP* and *PIF4-cYFP*. When *CRY1* was co-expressed with *TCP17-nYFP* and *PIF4-cYFP*, the fluorescence signals were significantly reduced and faded (Figure 5F). Consistently, the results from a ChIP experiment followed by PCR showed that under 22°C, loss of function of CRY1 (*cry1*) can significantly increase the binding affinity of TCP17 to the G-boxes in the promoters of PIF4 target genes, similar to that from Col-0 grown under 28°C (Figure S4), indicating enhanced binding activity of TCP17 to PIF4. In summary, our data demonstrated that CRY1 represses the interaction between TCP17 and PIF4, leading to lower PIF4 activity in regulating the expression of its target genes.

## Discussion

In this study, we illustrate a molecular framework that TCP transcription factors act as positive regulators in thermomorphogenesis by promoting the function of PIF4 at both transcriptional and post-transcriptional levels (Figure 5G). The regulation of TCP17 by temperature is at multiple different levels. TCP17 protein shows a very low abundance at 22°C. Elevated ambient temperature can increase the stability of TCP17, resulting in the accumulation of TCP17. The activity of TCP17 is also regulated by temperature. At a lower temperature, CRY1 physically interacts with TCP17 and inhibits not only the transcriptional activity of TCP17 but also the interaction between TCP17 and PIF4, leading to greatly reduced mRNA abundance and transcriptional activity of PIF4. The elevated ambient temperature can suppress the interaction between TCP17 and CRY1. Subsequently, TCP17 promotes the expression of *PIF4*, and the interaction between PIF4 and TCP17 is enhanced, leading to increased transcription activity of PIF4 toward its downstream thermoresponsive genes. Our results not only demonstrated novel roles of TCPs in regulating thermomorphogenesis but also proved that CRY1 can negatively regulate thermoresponse not only by directly inhibiting the transcription activity of PIF4 but also by repressing the activities of its positive regulators, like TCPs. These results contribute to our better understanding of the regulatory mechanisms of higher temperature on plant growth and development. The knowledge can be used for future crop improvements via molecular breeding or genetic engineering for higher productivity under a wide range of temperatures.

### Limitations of the Study

In this study, we demonstrated the important role of TCP transcription factors in regulating thermoresponsive hypocotyl growth. The stability of TCP17 protein is greatly increased at high temperature, the detailed mechanism of which is still unknown. In addition, as a temperature sensor, PHYB regulates thermomorphogenesis by repressing the activity of PIF4. Whether TCPs are involved in PHYB-mediated thermoresponses will be a very interesting research direction.

## Methods

All methods can be found in the accompanying Transparent Methods supplemental file.

## Supplemental Information

Document S1. Transparent Methods, Figures S1–S4, and Table S1
