# Supplementary material for: TCP Transcription Factors Associate with PHYTOCHROME INTERACTING FACTOR 4 and CRYPTOCHROME 1 to Regulate Thermomorphogenesis in Arabidopsis thaliana
Source: iScience. 2019 May 8;15:600–10. doi: 10.1016/j.isci.2019.04.002 (PMC6547012; doi:10.1016/j.isci.2019.04.002)
Supplement: Document S1. Transparent Methods, Figures S1–S4, and Table S1 [file mmc1.pdf]

ISCI, Volume *15*

Supplemental Information

TCP Transcription Factors Associate with PHYTOCHROME INTERACTING FACTOR 4 and CRYPTOCHROME 1 to Regulate Thermomorphogenesis in *Arabidopsis thaliana*

Yu Zhou, Qingqing Xun, Dongzhi Zhang, Minghui Lv, Yang Ou, and Jia Li

Supplemental Information

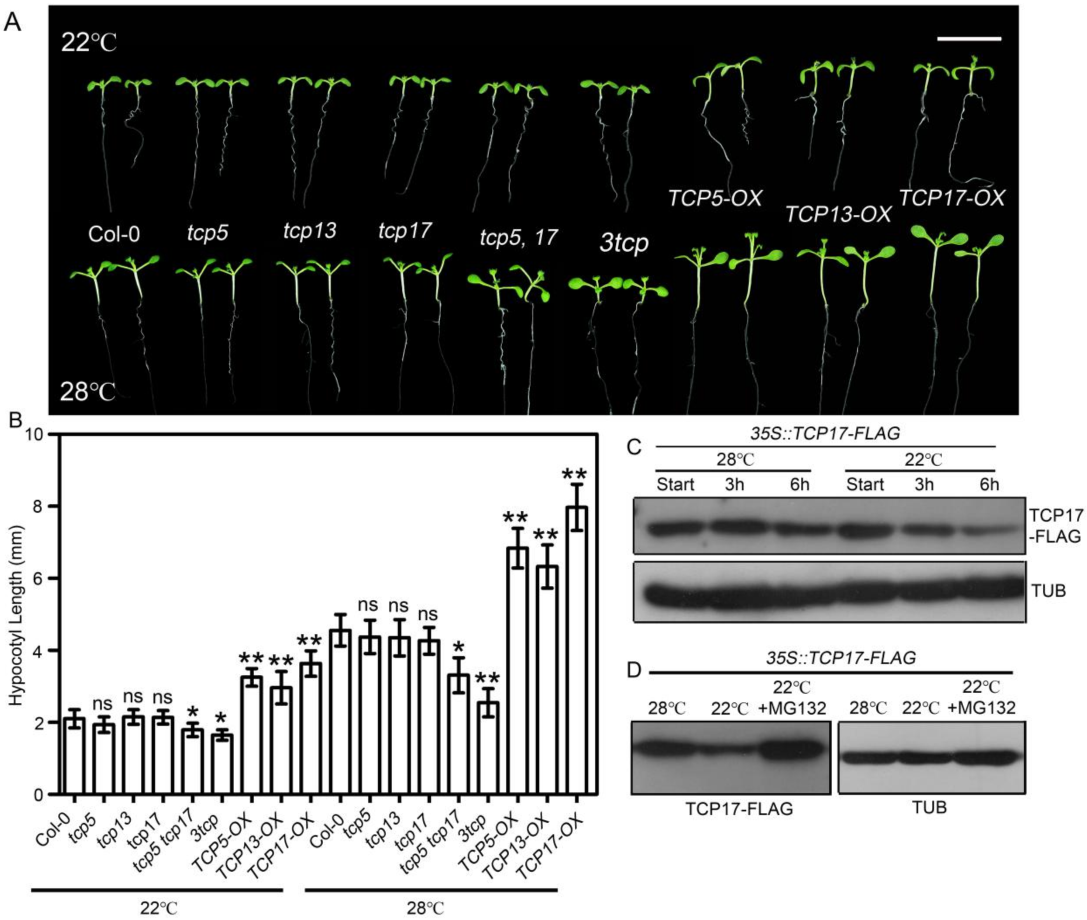

Figure S1. Related to Figure 1. The redundantly role of TCPs in regulating thermomorphogenesis positively. (A) Phenotypes of Col-0, *tcp5*, *tcp13*, *tcp17*, *tcp5 tcp17*, *3tcp*, *35S::HA-TCP5* (*TCP5-OX*), *35S::TCP13-GFP* (*TCP13-OX*), and *35S::TCP17-FLAG* (*TCP17-OX*) grown at 22°C and 28°C conditions. Scan bars represent 1 cm. (B) Measurements of the hypocotyl length shown in (A). Data shown were average and SEM (n≥20). ns p>0.05, *p< 0.05 and **p < 0.01; based on student's t test, which was performed by comparing each mutant to Col-0 grown under the same temperature. (C) The protein level of TCP17 was greatly reduced at lower temperature. Seven-day-old *35S::TCP17-FLAG* seedlings grown under LD, 22°C, were pretreated at 28°C for 24 hours and then transferred to 22°C or remained at 28°C for 3 or 6 hours. (D) The degradation of TCP17 protein at lower temperature was repressed by MG132. 28°C pre-treated *35S::TCP17-FLAG* seedlings were transferred to 1/2 MS media with or without MG132 and incubated at 22°C for 6

hours, as control, *35S::TCP17-FLAG* seedlings were treated at 28°C in 1/2 MS media without MG132 for 6 hours. In (C) and (D), immunoblot was carried out to detect the level of TCP17-FLAG and Tubulin by using an anti-FLAG or an anti-tubulin antibody, respectively.

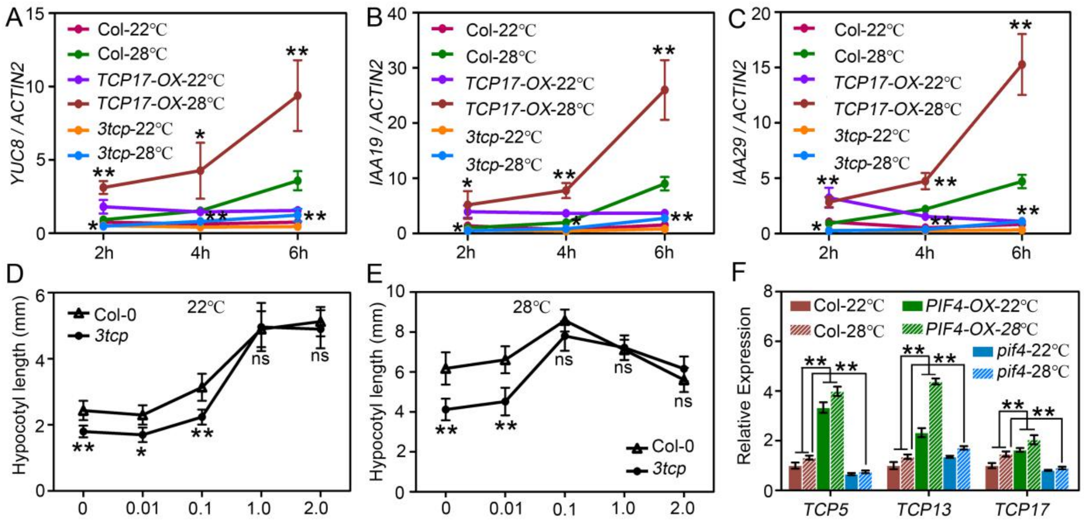

Figure S2. Related to Figure 2. TCPs play important roles in regulating PIF4-mediated thermoresponses.

(A-C) The responses of PIF4 targeted genes from Col-0, *TCP17-OX*, or *3tcp* to elevated temperature. Seven-day-old seedlings grown under 22˚C, LD condition were transferred to 28˚C or kept at 22˚C for 2, 4, and 6 hours before being collected for RNA extraction and real time PCR analyses. Data shown are the average and SEM of three biological replicates. *p< 0.05 and **p < 0.01; based on the student's t test, showing the comparison of *TCP17-OX*-28˚C or *3tcp*-28˚C to Col-28˚C. (D, E) PIC can rescue the short hypocotyl phenotype of *3tcp*. Col-0 and *3tcp* seedlings were grown under LD condition at 22°C with various concentration of an auxin analog, picloram (PIC), for 5 days and then transferred to 22°C (D) or 28°C (E) for additional 3 days before measurement was taken. Data shown were average and SEM (n≥20). ns p>0.05, *p< 0.05 and **p < 0.01; based on the student's t test. (F) The expression of *TCP5*, *TCP13*, and *TCP17* from Col-0, *PIF4-OX*, and *pif4* in response to elevated temperature. Seedlings were grown under LD, 22°C for 7 days, and then were transferred to 28°C or remained at 22°C for 4 hours. Data shown are the average and SEM of three biological replicates. *p< 0.05 and **p < 0.01; based on the student's t test.

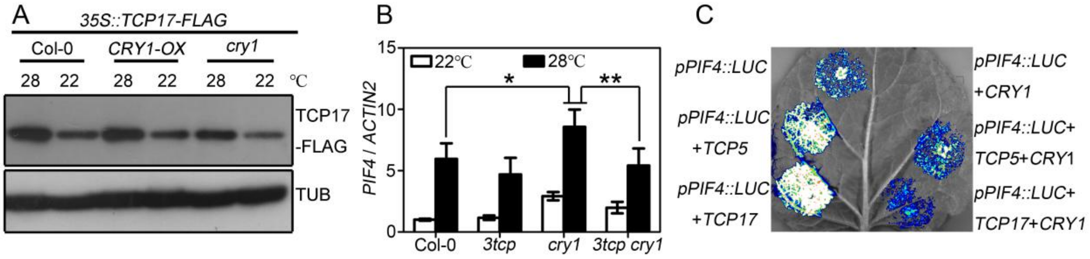

Figure S3. Related to Figure 5. CRY1 inhibits the activity of TCP17 to repress the expression of *PIF4*. (A) The response of TCP17 protein to temperature changes are not altered by CRY1. Seven-day-old *35S::TCP17-FLAG*, *CRY1-OX*/*35S::TCP17-FLAG*, *cry1*/*35S::TCP17-FLAG* seedlings grown under 22°C were pre-treated at 28°C for 1 day, and then transferred half of them to 22°C for 3 hours before being collected for protein extraction. Immunoblotting was taken out to detect the TCP17-FLAG protein level by using an anti-FLAG antibody. Tubulin probed by an anti-TUB antibody was used as an internal control. (B) The expression level of *PIF4* from Col-0, *3tcp*, *cry1* and *3tcp cry1* in response to elevated temperature. Seedling were grown under LD, 22°C condition for 7 days, and then were transferred to 28°C or remained at 22°C for 4 hours before being collected for analyses. Data shown were average and SEM. *p< 0.05 and **p < 0.01; based on student's t test. (C) CRY1 inhibits the transcriptional activity of TCP5 and TCP17 in a transient assay using *N. benthamiana* leaves. *pPIF4::LUC* was co-expressed with *35S::CRY1-HA*, *3S::TCP5-HA*, *35S::TCP17-FLAG*, *3S::TCP5-HA* with *35S::CRY1-HA*, or *35S::TCP17-FLAG* with *35S::CRY1-HA*, respectively. The luciferase activities were imaged 48 hours after co-infiltrated.

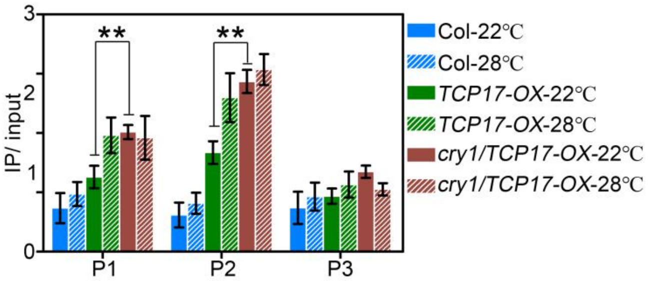

Figure S4. Related to Figure 5. CHIP assay on the promoters of *YUC8* and *IAA19* from Col-0, *TCP17-OX* (*35S::TCP17-FLAG*), and *cry1/TCP17-OX* (*cry1-35S::TCP17-FLAG*).

Seedlings were grown under LD, 22°C for 10 days, and then half of them were transferred to 28°C for 4 hours before collected for CHIP. P1, P2, and P3 are described as in Figure 3E and 3F. Data shown are the average and SD. *$p < 0.05$ and **$p < 0.01$. Student's t tests were used for the statistical analyses.

Table S1. Related to Figure 1, Figure 2, Figure 3, and Figure 4. Primer sequences used in this study.

	**Primers for Q-RT PCR analysis**
Actin2-F	TCAGATGCCCAGAAGTGTTGTTCC
Actin2-R	CCGTACAGATCCTTCCTGATATCC
TCP17-Q-F	TCTGGTAACGTCACTGTCGC
TCP17-Q-R	ACCACCACCGAGAAACGAAG
TCP5-Q-F	TCCTACTCCTCCGGCAATGA
TCP5-Q-R	AAGAGCTGAAGATGACCGGC
TCP13-Q-F	TTAGGGTTTCACGCGCCTTT
TCP13-Q-R	GCTGAATAGCCGTTGGGACT
PIF4-Q-F	TGCATCACAACCGACCGTAA
PIF4-Q-R	TGCTCGACTCCTTCGGTTTG
YUC8-Q-F	TGTATGCGGTTGGGTTTACGAGGA
YUC8-Q-R	CCTTGAGCGTTTCGTGGGTTGTTT
IAA19-Q-F	GGTGACAACTGCGAATACGTTACCA
IAA19-Q-R	CCCGGTAGCATCCGATCTTTTCA
IAA29-Q-F	TCCGATTTGAACGCCTATCCT
IAA29-Q-R	ACCGTGTGCATATACAAGATGTTTG
	**Primers for ChIP-qPCR analysis**
P1-F	atcgttgctccctaattcca
P1-R	TTTCTACCGACCATTTTTGT
P2-F	GATATCAAATGACTCCACGTGTC
P2-R	TCCGTGAAAGCTCTCTTCTTCAT
P3-F	GGTGCTACAAATGTCGTTTGGTA
P3-R	GATGGGGTTTAGAGGACGTAGGG
	**Primers used for cloning**
TCP5-F	AAAAAGCAGGCTTC ATGAGATCAG GAGAATGTGA
TCP5-R	AGAAAGCTGGGTC TCAAGAATCTGATTCATTATCGC
TCP5-NR	AGAAAGCTGGGTCAGAATCTGATTCATTATCGC
TCP13-F	AAAAAGCAGGCTTC ATGAATATCG TCTCTTGGAA
TCP13-R	AGAAAGCTGGGTC TCACATATGGTGATCACTTCCTC

TCP13-NR	AGAAAGCTGGGTCCATATGGTGATCACTTCCTC
TCP17-F	AAAAAGCAGGCTTC ATGGGAATAA AAAAAGAAGA
TCP17-R	AGAAAGCTGGGTCCTACTCGATATGGTCTGGTTGT
TCP17-NR	AGAAAGCTGGGTCCTCGATATGGTCTGGTTGT
PIF4-F	AAAAAGCAGGCTTCATGGAACACC AAGGTTGGAG
PIF4-R	AGAAAGCTGGGTCCTAGTGGTCCAAACGAGAACCGT
PIF4-NR	AGAAAGCTGGGTCGTGGTCCAAACGAGAACCGT
proTCP17-F	AAAAAGCAGGCTTC aatatggtccggtttgcagtgg
proYUC8-F	AAAAAGCAGGCTTC ATCCGATATGATAACGAT
proYUC8-R	AGAAAGCTGGGTC TGGAAGTTGTATTGGAAA
CRY1-F	AAAAAGCAGGCTTC ATGTCTGGTT CTGTATCTGG
CRY1-R	AGAAAGCTGGGTC TTACCCGGTTTGTGAAAGCCGTC
CRY1-NR	AGAAAGCTGGGTC CCCGGTTTGTGAAAGCCGTC
proPIF4-F	AAAAAGCAGGCTTCgatatggtccattacaagtaggcac
proPIF4-R	AGAAAGCTGGGTC GTCAGATCTCTGGAGACATTTCA

Transparent methods

Plant materials and growth condition

All the plants used in this study are Columbia accession. *tcp5* (CS116350), *tcp13* (CS313854), *tcp17* (*SALK_148580*), *pif4* (SALK_140393), and *cry1* (SALK_069292) were obtained from ABRC. *tcp5 tcp17* double mutant was generated by crossing *tcp5* with *tcp17*. *3tcp* triple mutant was obtained from Yuval Eshed's lab and was previously described (Efroni et al., 2008; Zhou et al., 2018). For constitutive overexpression, full length coding sequences of *TCP5*, *TCP13*, *TCP17*, *PIF4*, *CRY1*, and were cloned into *pEarley Gate201* (*35S::HA-TCP5* and *35S::HA-TCP13*), *pBIB-BASTA-35S::GWR-FLAG* (*35S::TCP17-FLAG* and *35S::PIF4-FLAG*), *pBIB-HYG-35S::GWR-GFP* (*35S::PIF4-GFP*, and *35S::TCP17-GFP*), and *pBIB-HYG-35S::GWR-HA* (*35S::CRY1-HA*) using a Gateway cloning approach. For *proTCP17::TCP17-GFP* transgenic plants, promoter region (1500 bp upstream of ATG) and coding sequence of *TCP17* amplified from genomic DNA was cloned into *pBIB-BASTA-GWR-GFP* by the Gateway cloning approach. *pif4/35S::TCP17-FLAG* was obtained by crossing *pif4* with *35S::TCP17-FLAG*. *3tcp/35S::PIF4-FLAG* plants were generated by crossing *3tcp* with *35S::PIF4-FLAG*. *3tcp cry1* quadruple mutant was obtained by crossing *3tcp* with *cry1*. *CRY1-OX*/*TCP17-OX* plants were generated by crossing *35S::TCP17-GFP* with *35S::CRY1-HA*. *35S::CRY1-HA*/*35S::TCP17-FLAG* plants were generated by crossing *35S::CRY1-HA* with *35S::TCP17-FLAG*. *35S::PIF4-GFP*/*35S::TCP17-FLAG* plants were generated by transforming *35S::PIF4-GFP* into a *35S::TCP17-FLAG* background. All the plants were grown in a greenhouse set at 22°C and a long day condition (LD, 16h light/8h dark) for general growth and seed harvesting.

Hypocotyl measurements

Surface-sterilized seeds were planted on 1/2 Murashige and Skoog (MS) medium containing 1 % sucrose and 0.8 % agar. For PIC treatment, various concentrations of PIC were mixed in the 1/2 MS medium. After 2 day vernalization, the plates were

placed under LD, 22°C condition for 5 days and then transferred to LD, 28°C or remained at LD, 22°C for additional 3 days before being harvested for analyses. Hypocotyls of seedlings were scanned and Image J software was used to quantify hypocotyl lengths. At least 20 seedlings were measured for each independent experiment. At least three biological replicates were carried out for each quantitative analysis.

RNA extraction and real time PCR

Seedlings were grown under 22°C, LD condition for 7 days, and then were transferred to 28°C or remained at 22°C for different time periods. Total RNAs were extracted using a Plant Total RNA extraction kit (Tiangen), and 1 μg total RNA was used for the first-strand cDNA synthesis using an Invitrogen reverse transcriptase kit, according to the manufacturer's instructions. Real time PCR was performed by using SYBR Premix Ex Taq II (TaKaRa) on an Applied Biosystems Step One Plus Real-time PCR system. The relative expression shown was the mean from 3 biological replicates after normalized against *ACTIN2*.

Immunoblotting

Protein extraction and immunoblotting were carried out as described previously (Zhou et al., 2018). *proTCP17::TCP17-GFP* and *35S::TCP17-FLAG* were grown under LD, 22°C condition for 7 days, then were treated for various time periods under different temperature conditions, before the whole seedlings being collected for protein extraction. The protein levels of TCP17-GFP and TCP17-FLAG were detected with an anti-GFP (Roche) or an anti-FLAG antibody (Abmart) respectively. TUBULIN probed with an anti-tubulin antibody (sigma) was used for as an internal control. The experiments were repeated three times and the similar results were obtained. One of the representative results was shown.

Yeast two-hybrid assay

The full length of *TCP17* cDNA was cloned into a *pGADT7* vector and the various

fragments of PIF4 and CRY1 were cloned into the *pGBKT7* vector based on manufacturer's instructions (Clontech). The construct *pGADT7-TCP17* was transformed into Y187 yeast cells, and the constructs containing various fragments of PIF4 or CRY1 were transformed into Y_2H Gold yeast cells. After selected on a synthetic dropout medium without Leu (-Leu, for *pGADT7-TCP17*) or Trp (-Trp, for *pGBKT7-PIF4-dAD*, *pGBKT7-CCE*, and *pGBKT7-PHR*), the yeast cells harboring *pGADT7-TCP17* were matted with yeast cells harboring *pGBKT7-PIF4-dAD*, *pGBKT7-CCE*, or *pGBKT7-PHR* for 24 hours, and then grown on synthetic dropout medium without Leu and Trp (-Leu/ Trp). Tree clones of each plate were picked up and grown on the synthetic dropout medium without Leu, Trp, His, and Adnine (-Leu/Trp/His/Adnine) containing 25 mM Aureobasidin A (ABA) to detect the interactions between TCP17 and PIF4 or CRY1.

BIFC

The full length cDNA of *TCP17*, and *TCP5* were cloned into *pEarley Gate201-nYFP*, while PIF4, CRY1 was cloned into *pEarley Gate202-cYFP*. *Agrobacterium* harboring each plasmid was incubated in LB medium containing 10 mM MES (PH 5.7) and 20 mM acetosyringone at 28°C overnight with shaking. After centrifugation, the pellets were resuspended in MS medium with 10 mM MES (PH5.7), 10 mM $MgCl_2$, and 150 mM acetosyringone to make a final concentration with OD_{600} up to 0.6. For co-transfections, equal volume of appropriate agrobacteria was mixed and the mixtures were incubated at room temperature for 3 hours before injection. After 48 hours of infiltration, the fluorescence was observed by a Leica confocal microscope.

Pull down assay

Full length coding sequence of TCP17 was fused in frame to the C terminus of the MBP tag by cloning into a *pMAL-cRI -GWR* vector. To make a FLAG-CRY1 fusion protein, CRY1 was cloned into the *pFLAG-MAC* vector by gateway method. For pull down assay, *E. coli* expressed MBP-TCP17 was purified using an amylose resin (NEB) following the manufacturer's instructions. The purified MBP-TCP17 protein was

incubated with the *E.coli*-purified FLAG-CRY1 protein under 4°C for 2 hours. After eluted using an elution buffer (10 mM maltose, 10 mM Tris-HCl, PH 7.5), the pull-down products were detected by immunoblotting using an anti-MBP or an anti-FLAG antibody.

Coimmunoprecipitation (co-IP)

Col and *35S::TCP17-FLAG* seedlings were used for detecting the interaction between TCP17 and CRY1, and for the interaction between PIF4 and TCP17, *35S::PIF4-GFP* and *35S::PIF4-GFP/35S::TCP17-FLAG* seedlings were used. Seven days old seedlings grown in a LD, 22°C growth chamber were transferred to 28°C or kept at 22°C for 4 hours before harvested for analyses. The tissues were grounded to fine powder in liquid nitrogen, and homogenized in IP buffer (50 mM Tris-HCl (PH 7.5), 1 mM EDTA, 75 mM NaCl, 0.5 % Triton X-100, 5 % Glycerol). After sonicated 5 times (10 seconds each time) with power output setting at 65 W, the extracts were centrifuged at 13,000 rpm for 15 min. The supernatant was mixed with 40 μl of anti-FLAG Affinity Matrix (SIGMA), and incubated at 4°C for 4 hours. The beads were washed 5 times with washing buffer (50 mM Tris-HCl (PH 8.0), 150 mM NaCl, 0.1 % Triton X-100). The bound proteins were eluted from the affinity beads with 2× SDS loading buffer boiled at 95°C for 10 min. The immunoprecipitation products were analyzed by immunoblot using an anti-FLAG, an anti-GFP, or an anti-CRY1 antibody.

Chromatin immunoprecipitation

Chromatin immunoprecipitation (ChIP) assays were performed as described previously (Ni et al., 2009). Col-0, *35S::TCP17-FLAG* and *cry1/35S::TCP17-FLAG* transgenic seedlings were grown in LD condition at 22°C for indicated time, and then transferred to 28°C or kept at 22°C for 4 hours. Two grams of plants were collected for ChIP assay. Chromatin was isolated and sonicated to generate DNA fragments with size ranging from 200 bp to 1000 bp. 40 μl of the anti-FLAG Affinity Matrix (SIGMA) were used for chromatin immunoprecipitation. Precipitated DNA was

analyzed by a real-time PCR. Three independent biological repeats were performed, and similar results were obtained. Relative fold enrichment shown in the results was the mean from one biological replicate after normalized against *ACTIN2*, and then against the respective input DNA samples. Student's t test was used for the statistical analyses.

Luciferase imaging

The transient expression assays in *N. benthamiana* leaves were carried out as previously described (Walley et al., 2007). The reporter *pPIF4::LUC* and *pYUC8::LUC* constructs were generated by inserting the promoter of *PIF4* or *YUC8* into the *pGWB235* binary vector by the Gateway cloning approach. *35S:TCP17-FLAG*, *35S::HA-TCP5*, *35S::PIF4-GFP*, and *35S::CRY1-HA* were used as effectors. The analyses were repeated three times, and similar results were obtained. Luciferase activities were imaged using a Lumazone CA 1300B camera.

Accession Numbers

TCP5 (AT5G60970), *TCP13* (AT3G02150), *TCP17* (AT5G08070), *PIF4* (AT2G43010), *CRY1* (AT4G08920), *YUC8* (AT4G28720), *IAA19* (AT3G15540), *IAA29* (AT4G32280).

Supplemental References

Efroni, I., Blum, E., Goldshmidt, A., and Eshed, Y. (2008). A protracted and dynamic maturation schedule underlies Arabidopsis leaf development. The Plant Cell *20*, 2293-2306.

Ni, Z., Kim, E.D., Ha, M., Lackey, E., Liu, J., Zhang, Y., Sun, Q., and Chen, Z.J. (2009). Altered circadian rhythms regulate growth vigour in hybrids and allopolyploids. Nature *457*, 327-331.

Walley, J.W., Coughlan, S., Hudson, M.E., Covington, M.F., Kaspi, R., Banu, G., Harmer, S.L., and Dehesh, K. (2007). Mechanical stress induces biotic and abiotic stress responses via a novel cis-element. PLoS Genetics *3*, 1800-1812.

Zhou, Y., Zhang, D., An, J., Yin, H., Fang, S., Chu, J., Zhao, Y., and Li, J. (2018). TCP Transcription Factors Regulate Shade Avoidance via Directly Mediating the Expression of Both *PHYTOCHROME INTERACTING FACTOR*s and Auxin Biosynthetic Genes. Plant Physiology *176*, 1850-1861.
